# Acute Basophilic Leukemia: Recent Molecular and Diagnostic Update

**DOI:** 10.7759/cureus.26054

**Published:** 2022-06-17

**Authors:** Gyanendra Singh

**Affiliations:** 1 Pathology, All India Institute of Medical Sciences, Rajkot, Rajkot, IND

**Keywords:** basophilic blast, bcr-abl fusion gene, gata1, myb, acute basophilic leukemia

## Abstract

Acute basophilic leukemia (ABL) is an uncommon subtype of acute leukemia characterized by clinical signs and symptoms related to hyper-histaminemia. Patients usually present with bone marrow (BM) failure due to the infiltration of BM by the blasts and may or may not have circulating blasts. Myeloid markers such as CD13 and CD33 are expressed by leukemic blasts, which are also positive for CD123, CD203c, and CD11b, but KIT (CD117) and other monocytic markers are usually negative. t(X;6) (p11; q23) translocation resulting in the MYB-GATA1 fusion gene has been seen in sporadic cases of ABL. Early phases of hematopoiesis are characterized by high levels of MYB and low levels of GATA1; as differentiation develops, an inverse regulation occurs, resulting in high levels of GATA1 and low levels of MYB.The translocation t(X;6) produces the MYB-GATA1 fusion gene (p11; q23). In mouse lineage-negative cells, MYB-GATA1 expression commits them to the granulocyte lineage and inhibited differentiation at an early stage. Cells expressing MYB-GATA1 show enhanced expression of markers of immaturity (CD34), granulocytic lineage (CD33 and CD117), and basophilic differentiation (CD203c and FcRI). NTRK1 and IL1RL1 transcription is directly triggered by MYB and MYB-GATA1, resulting in basophilic skewing of the blasts.

## Introduction and background

Joachim originally reported basophilic leukemia in two individuals with extreme basophilia and clinical symptoms of myelocytic leukemia in 1906 [1]. Wick et al. originally identified de novo acute basophilic leukemia (ABL) in 1982, and since then, many similar cases have been reported [2]. ABL is a rare type of acute leukemia that accounts for about 4-5% of all instances of acute nonlymphocytic leukemia [3,4]. It has a wide age range of occurrence and is associated with rapid clinical progression. The WHO 2008 classification has designated ABL as a discrete entity under the "acute myeloid leukemia (AML) not otherwise described" group, accounting for less than 1% of all AML cases [5]. Blasts ≥20% and immature basophils ≥40% of nucleated bone marrow (BM) or peripheral blood (PB) cells are the most recently proposed ABL criteria [6].

## Review

Clinical features

Patients usually present with BM failure as there is infiltration of BM by the blasts and may or may not have circulating blasts. Cutaneous involvement, organomegaly, and lytic lesions in the bone due to high histamine levels, such as pruritis and urticarial rash, can be seen. Gastroesophageal symptoms include nausea, vomiting, diarrhea, dyspepsia, and abdominal swelling or ulcers.

Morphology

**Figure 1:**
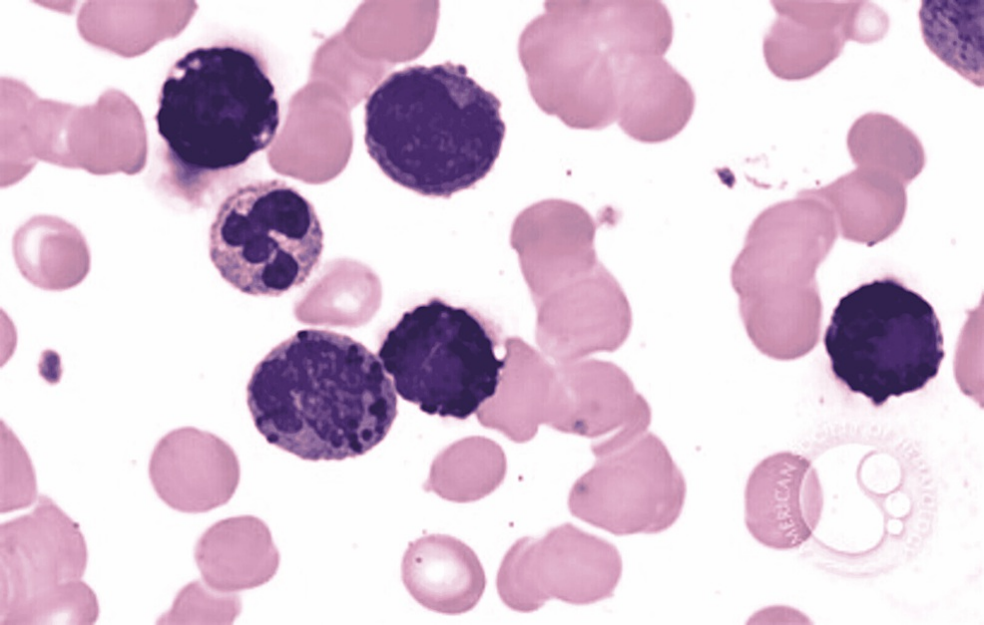
Basophilic blast with a round to oval nuclei with high nucleocytoplasmic ratio, prominent nucleoli, and a moderate amount of cytoplasm with coarse basophilic granules Image reference: Marion Eveillard and Alexandre Desjonqueres, Blood, 2014:123:3071

In ABL, the blasts have round- to oval-shaped nuclei with a high nucleocytoplasmic ratio, one to two prominent nucleoli with a moderate amount of cytoplasm, which can be either agranular or containing coarse basophilic granule. Mature basophils are less common (Figure 1).

Immunophenotype

Myeloid markers such as CD13 and CD33 are expressed by leukemic blasts, which are also positive for CD123, CD203c, and CD11b, but KIT (CD117) and other monocytic markers are usually negative. CD34 may be expressed by blasts. They may be positive for HLA-DR but negative for KIT, unlike normal basophils. Lymphoid-associated markers are usually negative [7,8].

The blasts of ABL are cytochemically negative for myeloperoxidase (MPO) and non-specific esterase (NSE). Metachromasia is seen with toluidine blue, and with AP, they show a diffuse reaction. The blasts of ABL usually express a myeloid phenotype and are positive for basophil-associated markers like CD9 and CD25 [9,10].

Both basophils and mast cells have myeloid phenotypes on immunophenotyping. Mast cells, on the other hand, are positive for CD117 but negative for CD25, whereas a reverse pattern is seen with mature basophils [11].

Genetic and molecular profile

However, because the disease is identified by morphology, there is currently minimal evidence on particular cytogenetic and molecular markers, but on the basis of recent research, more emphasis has been put on the MYB-GATA1 fusion gene. t(X;6) (p11; q23) translocation resulting in the MYB-GATA1 fusion gene has been seen in sporadic cases of ABL.

On human chromosome 6, the MYB gene codes for a 75-kDa transcription factor with a physiological role in the hematopoietic system. Early phases of hematopoiesis are characterized by high levels of MYB and low levels of GATA1; as differentiation develops, an inverse regulation occurs, resulting in high levels of GATA1 and low levels of MYB [12]. A gene on the human X chromosome codes for GATA1, a nuclear protein that plays an important role in hematopoiesis [13].

The translocation t(X;6) produces the MYB-GATA1 fusion gene (p11; q23). In mouse lineage-negative cells, MYB-GATA1 expression committed them to the granulocyte lineage and inhibited differentiation at an early stage. Cells expressing MYB-GATA1 showed enhanced expression of markers of immaturity (CD34), granulocytic lineage (CD33 and CD117), and basophilic differentiation (CD203c and FcRI) in a study conducted by Ducassou et al. [14]. NTRK1 and IL1RL1 transcription was directly triggered by MYB and MYB-GATA1, resulting in basophilic skewing of the blasts.

The presence of the Philadelphia chromosome, t(9;22) (q34; q11), in some cases of ABL shows that they were de novo cases of chronic myeloid leukemia (CML) with the preferential proliferation of mature and immature basophils [14]. t(3;6) (q21; p21) is another cytogenetic abnormality reported in ABL and abnormalities involving 12p AML with t(6;9) (p23; q34.1) are explicitly eliminated, as are instances associated with BCR-ABL1 [15,16]. A rare case of ABL with the t(16;21) (p11; q22)/FUSERG fusion gene has also been described in the literature [17].

Differential diagnosis

ABL should be differentiated from the following hematological entities:

(a) AML with t(6:9), as this leukemia has increased basophilic count [18].

(b) Blast crisis phase of CML has characteristic t(9:22) with specific clinical and hematological features [19].

(c) AML with BCR-ABL1 fusion gene (blasts express myeloid-associated marker but not the basophil-specific markers).

(d) Reactive increase in basophil counts.

(e) Acute promyelocytic leukemia with basophilic differentiation [18].

Prognosis and predictive factors

ABL is an extremely rare type of leukemia and very little information related to the prognosis and survival of this rare variant is available. But it has been observed that cases of ABL have a poor prognosis. Electron microscopy reveals that the granules contain an electron-dense particulate substance. These granules are internally bisected or contain crystalline material arranged in a lamellar pattern.

## Conclusions

ABL accounts for about 4-5% of all instances of acute nonlymphocytic leukemia, and it is associated with a wide age range of occurrence, rapid clinical progression, and poor outcomes. Patients usually present with BM failure, cutaneous involvement due to high histamine levels, organomegaly, and lytic bone lesion. Myeloid markers such as CD13 and CD33 are expressed by leukemic blasts, which are also positive for CD123, CD203c, and CD11b, but KIT (CD117) is negative. t(X;6) (p11; q23) translocation resulting in the MYB-GATA1 fusion gene has been seen in sporadic cases of ABL. ABL should be differentiated from AML with t(6:9), blast crisis phase of CML, AML with BCR-ABL1 fusion gene, and acute promyelocytic leukemia with basophilic differentiation.

## References

[1] Joachim G (1906). About mast cell leukemia (Article in German). Dtsch Arch Klin Med.

[2] Wick MR, Li CY, Pierre RV (1982). Acute nonlymphocytic leukemia with basophilic differentiation. Blood.

[3] Quattrin N (1978). Follow-up of sixty two cases of acute basophilic leukemia. Biomedicine.

[4] Peterson LC, Parkin JL, Arthur DC, Brunning RD (1991). Acute basophilic leukemia. A clinical, morphologic, and cytogenetic study of eight cases. Am J Clin Pathol.

[5] Arber DA, Brunning RD, Orazi A, Porwit A (2008). Acute myeloid leukaemia, not otherwise specified. WHO Classification of Tumours of Haematopoietic and Lymphoid Tissues.

[6] Valent P, Sotlar K, Blatt K (2017). Proposed diagnostic criteria and classification of basophilic leukemias and related disorders. Leukemia.

[7] Staal-Viliare A, Latger-Cannard V, Didion J, Grégoire MJ, Lecompte T, Jonveaux P, Rio Y (2007). CD203c/CD117-, a useful phenotype profile for acute basophilic leukaemia diagnosis in cases of undifferentiated blasts. Leuk Lymphoma.

[8] Staal-Viliare A, Latger-Cannard V, Rault JP (2006). A case of de novo acute basophilic leukaemia: diagnostic criteria and review of the literature (Article in French). Ann Biol Clin (Paris).

[9] Brunning RD, Matutes E, Borowitz M (2001). Acute myeloid leukemia. World Health Organization Classification of Tumours. Pathology and Genetics of Tumours of Haematopoietic and Lymphoid Tissues.

[10] Duchayne E, Demur C, Rubie H, Robert A, Dastugue N (1999). Diagnosis of acute basophilic leukemia. Leuk Lymphoma.

[11] Agis H, Füreder W, Bankl HC (1996). Comparative immunophenotypic analysis of human mast cells, blood basophils and monocytes. Immunology.

[12] Ramsay RG, Gonda TJ (2008). MYB function in normal and cancer cells. Nat Rev Cancer.

[13] Shimizu R, Engel JD, Yamamoto M (2008). GATA1-related leukaemias. Nat Rev Cancer.

[14] Ducassou S, Prouzet-Mauléon V, Deau MC (2017). MYB-GATA1 fusion promotes basophilic leukaemia: involvement of interleukin-33 and nerve growth factor receptors. J Pathol.

[15] Hoyle CF, Sherrington P, Hayhoe FG (1988). Translocation (3;6)(q21;p21) in acute myeloid leukemia with abnormal thrombopoiesis and basophilia. Cancer Genet Cytogenet.

[16] Hoyle CF, Sherrington PD, Fischer P, Hayhoe FG (1989). Basophils in acute myeloid leukaemia. J Clin Pathol.

[17] Toda Y, Nagai Y, Shimomura D (2017). Acute basophilic leukemia associated with the t(16;21)(p11;q22)/FUS-ERG fusion gene. Clin Case Rep.

[18] McKenna RW, Parkin J, Bloomfield CD, Sundberg RD, Brunning RD (1982). Acute promyelocytic leukaemia: a study of 39 cases with identification of a hyperbasophilic microgranular variant. Br J Haematol.

[19] Xue YQ, Guo Y, Lu DR (1991). A case of basophilic leukemia bearing simultaneous translocations t(8;21) and t(9;22). Cancer Genet Cytogenet.

